# The use of complementary and alternative medicine by patients with diabetes mellitus in Bahrain: a cross-sectional study

**DOI:** 10.1186/1472-6882-10-35

**Published:** 2010-07-14

**Authors:** Abeer J Khalaf, David L Whitford

**Affiliations:** 1 Department of Family & Community Medicine, Royal College of Surgeons in Ireland - Medical University of Bahrain, PO Box 15503, Busaiteen, Kingdom of Bahrain

## Abstract

**Background:**

CAM use is widespread, especially among patients with diabetes. The Gulf States have a high prevalence of diabetes, alongside a long tradition of CAM use. The aim of this study is to establish the prevalence of CAM use among patients with diabetes mellitus in Bahrain and to examine the characteristics of the CAM users.

**Methods:**

A questionnaire was developed and administered to a convenience sample of patients with diabetes (n = 402) above the age of 20 attending two hospital diabetes clinics. Data were analysed using descriptive statistics and non-parametric tests of association.

**Results:**

63% of responders utilized CAM within the previous 12 months. CAM users were more likely to be female, to have had diabetes for longer and to have complications of their diabetes. 64% of CAM users stated that they had used CAM for managing their diabetic condition, with 46% of these having used it solely for their diabetes. Respondents using CAM to manage their diabetes were more likely to be male, to be using CAM on a daily basis and to have informed their physician of their CAM use.

**Conclusions:**

There is a high rate of CAM use in patients with diabetes attending two hospital diabetes clinics in Bahrain. There is also a high rate of non-disclosure of CAM use to physicians. There is a continuing need for health professionals to be more aware and better trained in order to inform their decision making and communication related to CAM use.

## Background

The use of Complementary and Alternative Medicine (CAM) is widespread with a major reason for the use of CAM being the presence of chronic diseases such as diabetes mellitus[1]. CAM has been defined as the "practices, approaches, knowledge and beliefs incorporating plant, animal and mineral-based medicines, spiritual therapies, manual techniques and exercise"[2]. A recent review of CAM use in people with diabetes showed a wide variation in the prevalence of CAM use across nine countries from 17% to 73%[3]. This may be related to differences in the definition of CAM and varying research designs. However, the need for healthcare professionals to be aware of CAM use alongside conventional medicine remains.

The Gulf States have both a high prevalence of diabetes and a long tradition of CAM use. However, there has been little research carried out on the use of CAM in patients with diabetes in this region. A study in Saudi Arabia revealed a prevalence of CAM use for the management of diabetes of 30%[4]. The Kingdom of Bahrain has a similar high prevalence of diabetes to Saudi Arabia, ranking fourth in the world for the prevalence of diabetes[5]. The aim of this study is to establish the prevalence of CAM use among patients with diabetes mellitus in Bahrain and to examine the characteristics of the CAM users.

## Methods

### Questionnaire development

We developed a questionnaire to assess the use of CAM by patients with diabetes over the previous year. Questions were developed based on our research question and previous similar studies. In addition to asking about CAM use, questions explored the types of CAM used (based on the US National Centre for Complementary and Alternative Medicine (NCCAM) categorisation of CAM[6]), frequency of CAM use, benefits and problems with CAM, and CAM use in diabetes. A further question sought to establish whether patients using CAM disclose this use to their physicians. Background information was collected on the respondents' age, gender, educational status, ethnicity, diabetes and self reported diabetic complications. The questionnaires were translated into Arabic and piloted and refined.

### Sample

Diabetes registers were not available so the questionnaire was administered to a convenience sample of patients (n = 402) attending two hospital diabetes clinics. The Kingdom of Bahrain has a population of just over one million people, with approximately 40% being expatriates. Bahrain has a national health service with care being free at the point of contact. Bahrain does not have a fully developed shared care diabetes service. As a result hospital diabetes clinics care for a broad cross section of patients with diabetes. One of the diabetes clinics in the study was a government hospital, the other was a private hospital. The two hospitals serve a population of approximately 5000 patients with diabetes. Patients aged less than 20 years, those who did not speak Arabic or English, and those with dementia or any other form of limited understanding or learning difficulties were excluded.

### Data analysis

Data were analysed with JMP-IN v4, using both descriptive statistics and non-parametric tests of association.

Ethics approval was obtained from the RCSI-Bahrain research ethics committee, BDF Research Committee and Joslin Diabetes Center Research Committee.

## Results

402 questionnaires were returned. 18 patients did not meet the inclusion criteria, mainly on the grounds of language. 33 patients refused to participate. 252 (63%) responders had used CAM in the previous year. The relationship between CAM use and respondents' demographic and diabetes status is shown in Table 1. CAM users were more likely to be female, to have had diabetes for more than five years and to have complications of their diabetes (Table 1).

**Table 1:** Relationship between respondent characteristics and the prevalence of CAM use

Variable (n = 402)		Used CAM N (%)	Has not used CAM N (%)	Statistics
**Gender **(n = 402)	Male	103 (56%)	81 (44%)	X^2 ^= 6.53, 1 df, p = 0.01
		
	Female	149 (68%)	69 (32%)	

**Age **(n = 401)	20-39	42 (63%)	25 (37%)	X^2 ^= 3.18, 2 df, p = 0.20
		
	40-59	161 (66%)	84 (34%)	
		
	60 and above	49 (55%)	40 (45%)	

**Ethnicity **(n = 402)	Arab	232 (62%)	143 (38%)	X^2 ^= 1.61, 1 df, p = 0.21
		
	Non-Arab	20 (74%)	7 (26%)	

**Age finished full-time education **(n = 395)	Illiterate	25 (54%)	21 (46%)	X^2 ^= 4.83, 4 df, p = 0.31
		
	Less than 13 years	28 (65%)	15 (35%)	
		
	13-18	91 (69%)	41 (31%)	
		
	19-25	86 (63%)	51 (37%)	
		
	Over 25 years	20 (54%)	17 (46%)	

**Length of time with diabetes **(n = 400)	≤4 years	48 (49%)	50 (51%)	X^2 ^= 11.86, 3 df, p = 0.008
		
	5-9 years	66 (72%)	26 (28%)	
		
	10-15 years	69 (64%)	39 (36%)	
		
	≥16 years	68 (67%)	34 (33%)	

**Treatment of Diabetes **(n = 402)	Diet Only	7 (70%)	3 (30%)	X^2 ^= 3.04, 3 df, p = 0.39
		
	Diet & Tablets	140 (59%)	96 (41%)	
		
	Diet & Insulin	49 (65%)	26 (35%)	
		
	Diet, Tablets & Insulin	36 (59%)	25 (41%)	

**Complication of diabetes **(n = 402)	Present	130 (73%)	49 (27%)	X^2 ^= 13.63, 1 df, p = 0.0002
		
	Absent	122 (55%)	101 (45%)	

**Regular blood glucose monitoring **(n = 402)	Yes	141 (63%)	83 (37%)	X^2 ^= 0.02, 1 df p = 0.90
		
	No	111 (62%)	67 (38%)	

### Frequency of CAM use

43% of CAM users use CAM on a daily basis, with 8% using it at least once per month and the remaining 49% using it less frequently. Respondents using CAM on a daily basis were more likely to be using it for their diabetes (χ^2 ^= 40.78, 4 df, p < 0.0001), less likely to be Arabic (χ^2 ^= 8.21, 2 df, p = 0.016), more likely to report side effects of CAM (χ^2 ^= 9.61, 2 df, p = 0.008), and more likely to have informed their physician of their CAM use (χ^2 ^= 33.43, 2 df, p < 0.0001).

### CAM use for diabetes

161/252 (64%) CAM users stated that they had used CAM for managing their diabetic condition, with 46% of these having using it solely for their diabetes. Respondents using CAM specifically to manage their diabetes were more likely to be male (χ^2 ^= 7.4, 1 df, p = 0.006), to be using CAM on a daily basis (χ^2 ^= 31.05, 2 df, p < 0.0001) and to have informed their physician of their CAM use (χ^2 ^= 9.36, 1 df, p = 0.002).

### Type of CAM used

The majority of CAM users (227/252, 90%) used natural medicine as a form of CAM with 32% (n = 80) using alternative and medical practices, 10% (n = 24) using mind-body interventions, 31% (n = 78) using manipulative and body based methods and 3% (n = 8) using energy therapy as a form of CAM. Respondents using CAM to manage their diabetes were more likely to use natural medicine (χ^2 ^= 23.2, 1 df, p < 0.0001) but no more or less likely to use the other categories of CAM. Commonly used forms of natural medicine included garlic (36%), bitter melon (31%), cinnamon (30%) and fenugreek (27%).

### CAM use and conventional medicine

82% of CAM users (n = 207) state that they had found the use of CAM beneficial in managing their diabetes and/or other medical conditions. 14% of those who use CAM (n = 36) had encountered side effects from the CAM affecting the gastrointestinal, endocrine or nervous systems. The majority (87% (n = 217)) had used CAM along with prescribed medications such as metformin and aspirin. Only 38% (n = 95) of CAM users had disclosed their CAM use to their physician, whereas 45% (72/161) of those using CAM to manage their diabetes had informed their physician.

## Discussion

This study has shown a prevalence of CAM use of 63% amongst patients with diabetes attending two hospital clinics in Bahrain. The prevalence of CAM use in patients with diabetes in Bahrain is comparable with that in other similar studies in the USA (73%)[7], India (68%)[8], and Mexico (62%)[9], but higher than in Saudi Arabia (30%)[4], Australia (24%)[10], and the UK (17%)[10]. This may be related to different definitions of CAM and differing timeframes and we sought in this study to use the NCCAM categorization of CAM and examine use over the previous year.

Two thirds of CAM users had used CAM in the management of their diabetes, indicating that 40% of patients with diabetes in this study in Bahrain have used CAM in order to manage their diabetes. This is higher than in a previous study in Saudi Arabia[11], although they looked at use of traditional medicines alone. However 97% of respondents in this study used natural medicines as the form of CAM to manage their diabetes, suggesting that other factors are contributing to a higher use in Bahrain. Of note, it is not clear from the Saudi study as to the timeframe used to determine CAM prevalence and a shorter timeframe (less than a year as in this study) could account for some of the variation.

This study confirms an association shown in previous studies with longer duration of diabetes and the presence of complications[3]. This is not surprising as patients may seek to manage their diabetes and relieve complications proactively by using CAM after they have tried conventional medicine and found it to be inadequate. A study in the USA identified those aged over 65 years as being three times more likely to use CAM than those aged less than 65 years[12]. Other than age, a higher likelihood of CAM use has been shown to be associated with other factors such as ethnicity[13], a higher educational status[7,12] and blood glucose monitoring at home[10]. In this study, as in similar studies in Saudi Arabia[4,11], there was no association found between these factors and the use of CAM by patients with diabetes mellitus in Bahrain. This may be related to the relatively younger age of onset of diabetes in the populations in the Gulf States. However, as in the Saudi studies[4,11], we found an association between using CAM and being female. The cultural context and differing roles and health beliefs in the Gulf States between the genders may contribute to this.

It appears likely that regular CAM users (defined as daily use in this study) differ from those who use it occasionally. They are likely to be a more important group as they are more likely to suffer side effects and possible interactions with other medications. A particular concern has been the low disclosure rate of CAM use to physicians. This may be related to inadequate doctor-patient communication. The non-disclosure rate in this study of 62% falls within the range from other studies of 43-65%[9,12,14]. Of interest in this study is that regular users of CAM and those using CAM for the management of their diabetes (two groups at potentially higher risk) are more likely to inform their physician of their CAM use. There is little doubt that the use of both herbal medicine and conventional medicine can result in adverse effects from herb-drug interaction[15,16]. Therefore, a responsible healthcare approach is that patients should receive evidence based CAM information about efficacy, effectiveness, adverse effects and possible interactions, to inform their decision making related to CAM use.

A strength of this study is that it has examined CAM use in a population of very high prevalence of diabetes and has defined categories of CAM and timeframes. A weakness is that it was not possible to ascertain a random sample and caution needs to be exercised in generalizing conclusions from a convenience sample.

## Conclusions

There is a high rate of CAM use in patients with diabetes attending two hospital diabetes clinics in Bahrain. There is also a high rate of non-disclosure of CAM use to physicians. There is a continuing need for health professionals to be more aware and better trained in order to inform their decision making and communication related to CAM use.

## Competing interests

The authors declare that they have no competing interests.

## Authors' contributions

AJK conceived the study, participated in the design of the study and carried out data collection. DLW participated in the design and coordination of the study, performed the statistical analysis and drafted the manuscript. All authors read and approved the final manuscript.

## Pre-publication history

The pre-publication history for this paper can be accessed here:

http://www.biomedcentral.com/1472-6882/10/35/prepub
